# Elements of Anatomy

**Published:** 1838-01

**Authors:** 


Art. VII.—*Elements of Anatomy.* By Jones Quain, m.d. *Fourth Edition, revised and enlarged. Illustrated with Steel Plates and numerous Engravings on Wood.*—*London,* 1837. 8vo. Part I. pp. 491.

Of all the manuals of anatomy for the use of students that have yet come before us, Dr. Quain's is certainly the best; and the present edition, owing to the numerous and beautiful plates and woodcuts with which it is for the first time illustrated, is very superior to the preceding. In its present form, the work, when complete, will constitute a most elegant volume, and is calculated, no less for the practitioner who wishes to refresh his anatomical knowledge, than for the student and general reader.

